# The Palliation of Pediatric Kidney Transplantation

**DOI:** 10.1111/petr.70462

**Published:** 2026-09-16

**Authors:** Taylor R. House, Carrie Thiessen

**Affiliations:** ^1^ Division of Nephrology, Department of Pediatrics University of Wisconsin‐Madison School of Medicine & Public Health Madison Wisconsin USA; ^2^ Division of Transplantation, Department of Surgery University of Wisconsin‐Madison School of Medicine & Public Health Madison Wisconsin USA

**Keywords:** adherence, advance care planning, kidney transplant, palliative care, transition

## Abstract

To ease without curing—this is the essence of palliation. It is also, fundamentally, the objective of organ transplantation. A new kidney is not a cure but a bridge—a way to ease the symptoms of kidney disease and extend life. By definition, kidney transplantation is a form of palliative care. Yet, this alignment has been lost in some aspects of pediatric kidney transplant care. We examine four aspects of transplant care that reveal ongoing disconnection between patients, families, and clinicians across the transplant journey: the transplant lexicon, approaches to adherence, measures of success, and care transition. Applying core palliative care principles, like meaning, agency, connection, and hope, offers a necessary paradigm shift to bridge these clinical divides. To best serve transplant recipients and their families, the field of pediatric transplantation needs to reintegrate palliative care.

## 1 Introduction

Palliative care is the “active, total care” of a person with a life‐limiting or life‐threatening illness that is focused on alleviating the stress and symptoms of the illness and improving the quality of life for both the patient and their family [1, 2]. Derived from the Latin *palliare*, meaning “to cloak,” palliative care aims to shield patients from the sequelae of serious illness [3]. In this regard, the objectives of kidney transplantation are inherently palliative—to lessen the symptoms of chronic kidney disease (CKD), extend life, and ultimately enable people with CKD to flourish [4, 5]. Despite this functional alignment, clinicians often perceive palliative care as synonymous with care provided at the end of life and thus in conflict with the ethos of organ transplant [6, 7, 8, 9]. As a result, kidney transplant recipients and their families have historically missed out on the benefits afforded by palliative care [7, 10, 11, 12, 13, 14].

This disconnect exists in part because of the success of transplant medicine. Since the first kidney transplant just over 70 years ago, significant advances have transformed what was once a universally fatal condition into a manageable chronic state [15, 16]. This shift to long‐term chronic management is particularly evident in pediatrics. Five‐year survival rates of over 98% have been reported, with up to one‐third of grafts maintaining function at 20 years [4, 17, 18]. At the same time, these successes present new opportunities to affect outcomes like health‐related quality of life, care engagement, and priority alignment among pediatric kidney transplant recipients (pKTRs), their caregivers, and their clinicians [19, 20, 21, 22, 23, 24, 25]. Promoting pKTR flourishing—a state in which all aspects of life are good—requires re‐evaluation of kidney transplant through the lens of palliative care principles like meaning, agency, connection, and hope [5, 26, 27]. Specifically, the transplant lexicon, approaches to adherence, measures of success, and care transition represent four aspects across the transplant journey for a renewed palliative approach (Table 1).

**TABLE 1:** Aspects of pediatric kidney transplant care in need of palliative care reintegration.

Care aspect	Proposed palliative reorientation	Actionable strategies and tools
Transplant lexicon	Prioritize patient personhood, acknowledge dual tensions introduced by transplant, redefine normalcy on patient and family terms	– Employ objective descriptors like “the kidneys are not working” – Validate dual tensions inherent to transplant – Avoid describing aspects as “normal,” and promote discussions of what it is to lead a meaningful life
Approaches to adolescent and young adult (AYA) adherence	Develop AYA‐centered partnerships, prioritize AYA psychological needs, and support health behaviors through an intrinsically motivated, developmental approach like Self‐Determination Theory	– Co‐create care plans with AYAs—Eliminate adultist microaggressions in verbal and written communication—Design and implement developmentally grounded interventions that support self‐motivation
Measures of success	Enable holistic flourishing by investigating, incorporating, and prioritizing what matters most to patients and families	– Develop and routinely administer transplant‐specific quality of life scales—Customize care plans to match patient and family‐identified hopes and tradeoffs
Care transition	Emphasize transition as a longitudinal developmental milestone centered on cultivating agency, mitigating uncertainty, and processing the grief of fractured connections	– Rebrand advance care planning as an exercise of supportive autonomy striving for value‐aligned care—Adapt existing pediatric advance care planning tools for pKTRs—Utilize legacy tools—Leverage dually trained pediatric nephrology palliative care clinicians

*Note:* The transplant lexicon, approaches to adherence, measures of success, and care transition represent four aspects of kidney transplantation proposed for palliative reorientation. Practical recommendations for implementation are provided.

## 2 The Transplant Lexicon

“Your kidneys have failed, but a normal life is possible with the gift of kidney transplant.” A youth and family might hear something similar at the start of their transplant journey while undergoing evaluation. Yet while terms like “failure,” “normal,” and “gift” may seem innocuous and commonplace, the deeper meanings attached to them are potentially harmful. For the pKTR, these words do more than describe a clinical state; they actively shape an identity.

Adult patients with CKD and their caregivers have emphasized the negative psychological impact of describing kidneys as “failing” [28]. While kidney “failure” may be an objective event in clinical terms, the “failure” of one's organs may be difficult to separate from feelings of personal failure, ultimately reducing a patient to being defined by their disease [28]. This is compounded by the fact that other chronic diseases are not categorized this way. Tong et al. described the frustration of an adult with CKD, “No one says, ‘you've got diabetes, your pancreas has failed.’” [28] This linguistic burden can be increased for pKTRs experiencing loss of graft function. Because long‐term transplant survival is heavily framed as dependent upon a recipient's behaviors and habits, the “failure” of a transplant, especially from causes like rejection, is rarely viewed as a neutral physiological event. Instead, “failure” of a transplant can reinforce the implication of a personal shortcoming, contributing to psychological distress, poor mental health, and ultimately worse health outcomes [28]. This burden also extends beyond the patient [28, 29]. In pediatrics, where caregivers often bear primary responsibility for their child's care, parents may internalize transplant “failure” as a direct reflection of their parenting abilities and as fundamentally in conflict with what it means to be a “good parent” [30, 31, 32]. To mitigate these hidden harms, a deliberate shift toward palliative care principles is required. To preserve agency and personhood while still providing clear meaning, clinicians can pivot to objective, functional descriptors—explaining that the kidneys are “not working” or are “no longer able to do the job” of keeping a person alive. This linguistic shift re‐aligns the clinical narrative away from a personal deficit and disease‐centric model and toward a physiologic limit.

Similarly, “normal” is a nebulous, potentially problematic term often used to describe life after kidney transplant. Historically, normality has been the basis for comparison in medicine—“normal” labs, “normal” urine output, “normal” growth and development—and is at times treated as synonymous with “health” [33]. Yet, while kidney transplant offers the chance to “normalize” many of these values, many youth and families may perceive that there is nothing “normal” about taking medications every 12 h, drinking in excess of one's thirst, and living with the constant knowledge that a graft has a finite lifespan [34]. Feeling fundamentally different from peers because of these inevitable elements of transplant can give pKTRs and families the perception that something is wrong with them or their transplant course [34]. At the same time, achieving a sense of “normalcy” and fitting in with peers may remain a primary goal for youth and their families [35]. This can be further complicated by the fact that, for youth born with CKD, living with chronic illness *is* their “normal.” Emphasizing palliative care principles of meaning, agency, and hope allows a shift away from externally imposed definitions of normality toward normality defined by the patients and families themselves. Rather than assuring families a return to “normal” life after transplant, discussions must center on building a meaningful life within the constraints of chronic illness, exploring what being “normal” means to that unique youth and family, and embracing life on new terms as a pKTR [33].

This pressure to project an illusion of “normalcy” can be further compounded by the pervasive imagery of organ transplants as life‐saving “gifts” [36]. Though meant to honor the donor, describing a kidney transplant as a “gift” introduces a hidden psychological cost. When someone receives the “gift” of a kidney, it can impose an identity of one who is indebted to both the gift and gift giver [37, 38]. Gift terminology places pKTRs at risk of experiencing a “gratitude paradox” [39]. When transplant is framed as a “miracle,” or a “gift of life,” it creates an implicit obligation to be grateful, leading pKTRs to internalize the belief that they cannot simultaneously hold grief or dissatisfaction alongside gratitude [40]. Consequently, expressing expected frustrations, like distress over steroid‐induced weight gain or the exhaustion of rigid medication schedules, can be perceived as a betrayal of the donor's sacrifice. This narrative effectively silences patients, discouraging open discussions of the challenges inherent to living with a kidney transplant [39, 40]. Such emotional suppression can also be particularly notable in pediatric transplant, where the donor may be a living parent, fundamentally complicating family dynamics and boundaries [40]. Framing transplant as a critical medical tool or treatment that requires hard work to maintain can serve to acknowledge the value of organ donation without imposing expectations on pKTRs. If families use gift imagery, applying palliative principles of meaning and agency involves exploring the youth's unique perspective on receiving a transplant. Clinicians can intentionally create space to recognize these dual tensions, for example asking questions like, “What are the parts of having a new kidney you are glad about, and what are the parts that are still hard or frustrating?” This validates the full spectrum of pKTRs' lived experience, empowering them in their care while minimizing hidden psychological burdens. Ultimately, the words used during the earliest points in transplant care set the stage for the next phases in a pKTR's journey. The identities and expectations shaped by the transplant lexicon impact how a young person experiences the daily reality of life after kidney transplant.

## 3 Approaches to Adolescent and Young Adult Adherence

Perhaps the most unforgiving aspect of this daily reality is the demand for near‐perfect medication adherence, which remains a long‐standing hurdle in pediatric kidney transplant medicine [41, 42, 43]. While adherence is primarily caregiver‐driven for younger children, during adolescence it increasingly shifts to a patient‐directed task, and this transfer of responsibility can be precarious and determinative. Just as the transplant lexicon can inadvertently alienate patients and families, standard clinical approaches to adolescent and young adult (AYA) adherence often miss the mark. Applying a palliative lens provides a necessary paradigm shift, introducing innovative ways to conceptualize and optimize AYA adherence.

While AYA pKTRs face the highest risk of graft loss across the lifespan [43], they are concurrently at risk of having their agency threatened by age‐based microaggressions. Initially used to describe the “brief and commonplace daily verbal, behavioral, and environmental indignities” that communicate derogatory slights to marginalized groups [44], microaggressions in pediatric kidney transplant medicine are often rooted in adultism. Adultism is age‐based oppression that preferentially centers adult perspectives over those of AYAs, subsequently presenting them as incompetent or untrustworthy [45]. In transplant care, these microaggressions manifest when a care plan is developed without the input of the AYA, when a clinician documents in a note that a patient merely “reports” adherence, or when colleagues comment on rounds that, “She claims she never misses a dose, but her last tacrolimus level was mysteriously undetectable.” Such language both undermines therapeutic alliance and dismantles an AYA's credibility, competence, and expertise, stunting their development as a partner in their own care. While adult transplant recipients may face similar presumptions about their adherence, these clinical practices expose a unique double standard for AYAs—they are expected to implement complex medical regimens without missteps, but their perspectives are routinely excluded in care planning, and they are denied the benefit of the doubt granted to younger children whose care remains caregiver‐driven [46, 47, 48]. For AYAs who do struggle with non‐adherence, this adultist lens misrepresents their behavior as defiance, laziness, or apathy rather than an attempt to achieve an unexamined definition of “normal,” a predictable developmental struggle, or a sign of unmet needs for tangible support.

This systematic mischaracterization makes enhancing support increasingly challenging, ultimately contributing to the lack of effective adherence interventions [49, 50]. For example, though over 50 adherence interventions have been designed for adult kidney transplant recipients or AYA recipients of other solid organs, a Cochrane review found inadequate evidence to support universal implementation of any one intervention [51]. Of the three main interventions designed specifically for AYA pKTRs, only one significantly reduced rejection rates, and all three rely heavily on clinical and surveillance‐based strategies [52, 53, 54, 55]. Considering that this type of patrolling does not yield long‐term graft survival, a palliative approach suggests that adherence interventions must shift away from behavioral surveillance and toward a psychological and developmental grounding. Self‐Determination Theory offers a strong framework for this shift. This psychological model of intrinsic motivation rather than external compliance asserts that three basic psychological needs—autonomy (control over one's actions), competence (mastery of tasks and skills), and relatedness (emotional connection with others)—must be met for an individual to engage in their care and undertake health behaviors like adherence [56]. Beyond its direct alignment with palliative care principles, Self‐Determination Theory also heavily overlaps with core developmental tasks of adolescence including individuation, self‐sufficiency, and cultivation of relationships [57, 58]. Interventions based in Self‐Determination for AYAs living with other chronic conditions have already demonstrated significant improvements in health outcomes [59, 60]. Applying this framework to pKTRs holds high promise, transforming approaches to adherence from one of adult‐driven monitoring to youth‐centered partnership.

## 4 Measures of Success

This type of partnership cannot succeed until the metrics defining what it means to flourish reflect outcomes valued by patients and families. Success post‐transplant has historically been framed narrowly around patient and graft survival as reflected in peer‐reviewed publications, publicly available center metrics, and in the US, Centers for Medicare & Medicaid Services regulatory tracking. While these are critical outcomes, this focus omits other factors that patients and families may regard as equally or more important determinants of success, such as quality of life, number of hospital admissions and clinic appointments, and the ability to pursue life goals [61, 62]. Standard metrics also fail to account for the burden of lifelong immunosuppression. In one of the largest surveys of adult transplant recipients, patients identified pain, fatigue, weight gain, and weakness as the most burdensome side effects impacting daily life [63]. This mirrors findings among AYA pKTRs who rated pain and changes in appearance as the most distressing side effects [64]. In response, recent publications call for the development and longitudinal administration of transplant‐specific, patient‐centered quality‐of‐life scales [65, 66, 67]. Since patient and family‐centered perceptions of success may vary across developmental phases, such instruments may need to be modified and re‐validated for the pediatric population.

Clinicians also do not need to wait for population‐level research studies to redefine “success.” They can proactively integrate patient and caregiver voices at an individual level by asking pKTRs to share their priorities and hopes for their transplant care and developing customized care plans targeting these goals. For a pKTR with severe needle phobia, for example, this may require explicitly balancing the trade‐offs between the patient‐centered value of minimizing blood draws and the clinical objective of maximizing graft longevity while seeking supportive care from allied professionals.

## 5 Care Transition

The final milestone in the pediatric kidney transplant journey is transition to adult care. While transition is a longitudinal process beginning with caregiver‐supported preparation in childhood, the phase approaching active care transfer is a precarious period of vulnerability. Heightened family strain and pressure on AYAs to independently manage their care can contribute to accelerated CKD progression, trigger avoidable hospitalizations, and ultimately lead to graft loss [68, 69]. Transition interventions tend to focus on the logistics of care and patient education and have shown variable impacts on health outcomes [69]. Like adherence interventions, such operational frameworks are at risk of failure if they do not fully account for the profound developmental and psychological evolution inherent to this milestone. Beyond the transfer of clinical responsibility, applying a palliative lens reframes transition as a longitudinal process of cultivating agency, managing the grief of severing connections, and navigating the uncertainty of an unfamiliar medical system.

Specifically, advance care planning offers an underutilized framework for self‐advocacy and identity preservation to target increased agency throughout transition [70, 71, 72, 73]. Historically, models of advance care planning for adults have not consistently improved healthcare utilization or goal‐concordant care at the end‐of‐life [74]. This association with the end of life can also lead to the practice being overlooked or avoided. Yet, the overarching goal of advance care planning—promoting a shared discussion between a patient, their family, and clinicians to align their preferences and goals with future medical treatments—can be effectively applied to provide AYAs with concrete strategies to maintain a sense of control [70, 75]. The demand for such an approach is supported by data from other pediatric populations; AYAs with chronic conditions including cystic fibrosis, sickle cell disease, HIV infection, and cancer endorse a desire to engage in structured expressions of care preferences [70, 71, 72, 73]. While evidence‐based pediatric advance care planning tools like the Family/Adolescent Centered (FACE) intervention and *Voicing My Choices* offer a foundation to build upon, they may still be hampered by obstacles encountered in adult settings [72, 76]. In addition to being adapted for the unique needs of AYA pKTRs, these interventions may benefit from a conceptual rebranding emphasizing the objective of value‐aligned care. For example, rebranding palliative care in adult nephrology to “kidney supportive care” has helped to address misconceptions and clarify its ultimate purpose [77]. A similar shift could lessen stigma and reframe advance care planning as a tool to transform potential ambiguity into a proactive exercise of supported autonomy for pKTRs.

While advance care planning targets an AYA's agency and mitigates uncertainty, a parallel strategy is required to address the relational trauma of leaving pediatric care. Many pKTRs receive care from the same pediatric team for years and at times, since infancy. The bonds developed through the longitudinal depth of these relationships frequently lead youth to view their clinicians as extended family [78, 79]. As a result, severing these ties through transition can trigger profound feelings of rejection, isolation, loss, and disenfranchised grief [78, 79, 80]. Collectively, transition represents a milestone in a youth's healthcare “legacy,” conceptually defined as “the process of leaving something behind” [81]. Legacy interventions are patient and family‐centered activities designed to prompt meaning‐making and reflection on one's medical journey that are often incorporated into specialty palliative care delivery [82]. Among adults and children with life‐limiting and life‐threatening illnesses, legacy interventions have demonstrated improvements in emotional well‐being, self‐efficacy, quality of life, coping, and parent–child connection and communication [82, 83, 84]. While no transplant‐specific legacy interventions currently exist, tools like *My Kidney Identity*, visualizations, and photonarratives could be adapted for use at transition [34, 85, 86]. These creative modalities could provide transitioning AYAs with a structured, therapeutic way to reflect on their pediatric care, process the grief associated with fractured healthcare relationships, and look toward the opportunities of young adulthood [34, 85, 86].

Ultimately, effective utilization of tools like advance care planning and legacy work requires a clinical infrastructure backed by a collective recognition of the potential trauma and suffering associated with transition. To systematically meet this need, we have previously proposed the standard integration of pediatric nephrologists with additional palliative care expertise at inflection points in care, including transition [87]. Such dual‐trained kidney palliative care teams could serve as both a bridge to adult care while also attending to the emotional and relational distress related to transition.

## 6 Conclusion

While advances in transplant medicine have extended the lives of pKTRs, the daily reality of managing a kidney transplant places them at high risk of failing to flourish. Changes are needed across four key aspects of transplant care—adopting a transplant lexicon that preserves personhood; utilizing adherence strategies grounded in developmental theory; expanding measures of success to incorporate those prioritized by patients and families; and recognizing transition as an opportunity to cultivate agency and address grief. Applying a palliative care lens offers a framework to address these threats to well‐being and ultimately add life to the years a transplant affords [10, 88].

## Data Availability

The authors have nothing to report.
